# Metal Oxide Semiconductor Gas Sensors for Amine-Based Carbon Capture: A Mini Review

**DOI:** 10.3390/ijms27219596

**Published:** 2026-10-27

**Authors:** Yi Ye, Wenqi Fan, Longjie Liu, Cengjun Ning, Liang Huang, Xinglei Zhao, Jiale Zheng

**Affiliations:** 1 CNPC Research Institute of Safety and Environmental Technology, Beijing 102206, China; yeyi@cnpc.com.cn (Y.Y.); fan-wenqi@cnpc.com.cn (W.F.); liulongjie@cnpc.com.cn (L.L.); ningchenjun@cnpc.com.cn (C.N.); zhaoxinglei@cnpc.com.cn (X.Z.); 2 State Key Laboratory of Petroleum Pollution Control, Beijing 102206, China; 3 College of Environmental Science and Engineering, Beijing Forestry University, Beijing 100083, China; l_huang@bjfu.edu.cn

**Keywords:** carbon capture, post-combustion amine capture, MOS gas sensors, MEA detection

## Abstract

Carbon capture, utilization and storage (CCUS) is indispensable for achieving global carbon neutrality, and amine-based chemical absorption remains the most mature and widely deployed technology. However, amine emissions during operation result in absorbent loss, secondary pollution and potential public health risks, while conventional offline detection methods are unable to satisfy the requirements of real-time monitoring. This review systematically summarizes recent progress in metal oxide semiconductor (MOS) gas sensors for monoethanolamine (MEA) detection, with particular emphasis on AOx-based systems. The effects of the base material, microstructure and modification strategies, including noble metal decoration and heterojunction construction, on sensing performance are critically compared. Through such modifications, AOx-based sensors have achieved markedly improved sensing performance, and several are capable of detecting MEA at room temperature. The feasibility of existing sensing materials is further evaluated in the context of post-combustion amine capture. Among the reported systems, SnO_2_-based sensors exhibit clear advantages for MEA detection, including high sensitivity, favorable selectivity, rapid response and recovery, and satisfactory stability and repeatability. In contrast, studies on ABO_3_ perovskite-based sensors remain limited; although these materials show fast response, they continue to suffer from poor stability and insufficient humidity resistance. Future research should therefore focus on low-temperature operation, enhanced humidity tolerance and long-term durability, so as to accelerate the industrial translation of MOS gas sensors and support the sustainable development of CCUS.

## 1. Introduction

The large-scale anthropogenic emissions of greenhouse gases, dominated by carbon dioxide (CO_2_), have resulted in the persistent intensification of the greenhouse effect. Global warming, and the diverse extreme weather events it triggers, pose an increasingly grave threat to the survival and sustainable development of human society [1,2,3]. In 2024, leading international authorities, including the Copernicus Climate Change Service and the World Meteorological Organization, jointly confirmed that the global average temperature increase had exceeded the 1.5 °C long-term warming threshold set by the Paris Agreement for the first time, with the annual average temperature calculated from multi-source observational data reaching 1.55 °C [4]. This landmark milestone fully underscores the extreme urgency of accelerating carbon emission reduction initiatives worldwide.

**Figure 1:**
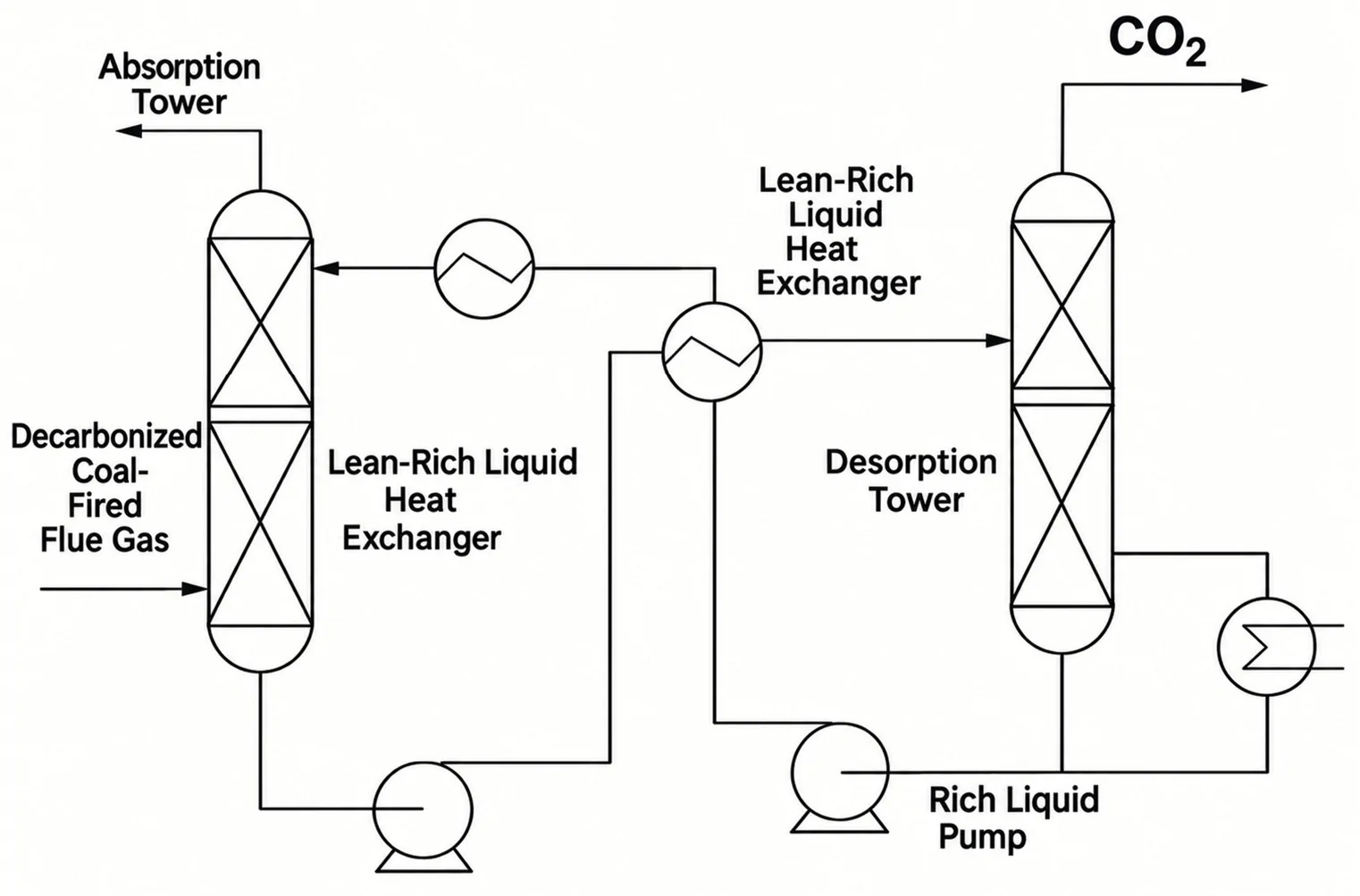
Schematic of a typical CO_2_ chemical absorption capture system.

Carbon capture, utilization and storage (CCUS) is widely recognized as an indispensable core technological pathway for achieving the global carbon neutrality goal at the current stage [5]. Carbon dioxide (CO_2_) capture refers to the process of separating high-purity CO_2_ from flue gas generated by the combustion of fossil fuels and biomass fuels in the power and industrial sectors, or directly capturing CO_2_ from the atmosphere [6]. Chemical absorption using organic amine solutions is currently the most mature and widely adopted mainstream technology in the field of carbon capture (as shown in Figure 1), and has been deployed for large-scale engineering applications in major emission-intensive industries such as power, chemical engineering, and iron and steel. Typical engineering cases include the 1 million t/a CO_2_ capture project at Boundary Dam Coal-Fired Power Station in Canada [7], the 1.4 million t/a CCS project of Petra Nova [7], the 120,000 t/a carbon capture demonstration project at Huaneng Shanghai Shidongkou No. 2 Power Plant, the 150,000 t/a full-process CCUS demonstration project at Shenhua Guohua Jinjie Power Plant [8], the 500,000 t/a carbon capture project at Jiangsu Taizhou Power Plant of China Energy Investment Corporation [9], and the 1.5 million t/a CCUS demonstration project at Huaneng Zhengning Power Plant [10]. Nevertheless, during the capture process, where absorbents come into direct contact with flue gas, parts of the amine-based absorbents and their degradation products are discharged along with purified flue gas in the form of gas or aerosols [11]. Such flue gas pollutants feature large total emissions and are accompanied by aerosol formation, bringing enormous challenges to pollution control. This issue not only leads to absorbent loss and reduction in system capture efficiency, but also gives rise to potential risks of secondary environmental pollution [12].

During chemical absorption, CO_2_ reacts with amine groups (-NH_2_) to form carbonates or bicarbonates. This process can be represented by the following reaction equation:

CO_2_ + H_2_O + 2RNH_2_←→(RNH_3_)_2_CO_3_

Taking the most commonly used absorbent, MEA, as an example, during the continuous operation of chemical absorption systems, the solvent loss caused by flue gas emissions into air can reach 0.01~0.8 kg per ton of captured CO_2_ [13,14]. Meanwhile, the emitted exhaust gas also contains pollutants, such as volatile degradation products of absorbents (represented by ammonia) and secondary particulate matter. When conducting environmental impact assessments of chemical absorption systems, the Mongstad Technology Center in Norway has set the organic amine emission limit for CO_2_ capture systems at 6 ppm [15]. However, field measurement data from multiple domestic and international research institutions indicate that the organic amine emission concentrations of actual operating chemical absorption systems generally range from 20 to 700 ppm [16,17,18,19], far exceeding the aforementioned limit. Once released into the atmosphere, these pollutants can further form highly toxic substances such as nitrosamines and nitramines through photooxidation reactions or chemical reactions with rainwater, some of which have been classified as potent carcinogens [20,21,22]. Such toxic substances can enter the human body through respiratory inhalation, skin contact, and other routes, posing a serious threat to public health [23].

The required purity of captured CO_2_ and the stringency of monitoring depend largely on its final utilization pathway. In agricultural and food-related applications, ultra-high CO_2_ purity is essential, since even trace amines are unacceptable owing to their potential phytotoxicity and the strictness of food safety regulations, which makes high-precision monitoring indispensable. In chemical synthesis, residual amines may act as catalyst poisons and thereby compromise process efficiency and product quality, so rigorous monitoring is likewise required. In enhanced oil recovery, the main concerns associated with amine carryover are pipeline corrosion and potential environmental leakage.

According to the Net Zero Emissions by 2050 Scenario released by the International Energy Agency (IEA), the global carbon capture capacity needs to reach 1.67 billion t/a by 2030 and further rise to 7.6 billion t/a by 2050 [24]. In its publications Reaching Zero with Renewables [25] and Renewable Energy Outlook [26], the International Renewable Energy Agency (IRENA) projects that carbon capture will contribute around 2.79 billion tons of CO_2_ removal annually by 2050. At this development scale, the annual amine emissions from the global carbon capture industry will range from 10 thousand to millions of tons after 2030. When these pollutants enter the atmosphere, water bodies and soil, they will exert long-term and far-reaching adverse impacts on the global ecosystem. Therefore, the regulation of amine emissions from CCUS projects will become an inevitable requirement for industrial development in the future. Meanwhile, high-precision and high-reliability online monitoring technologies for amines will embrace a strategic opportunity for large-scale application and rapid development.

This paper systematically reviews the research progress of amine online monitoring technologies, the emission characteristics of carbon capture processes, and the future development trends in this field. It also conducts an in-depth analysis of the working principles and technical limitations of existing monitoring technologies. The purpose of this paper is to clarify the practical engineering requirements for the online monitoring of gaseous pollutant emissions during carbon capture processes. By systematically classifying the current research status and core technical challenges in this field, this work provides a theoretical basis and technical reference for the research, development, and engineering application of pollutant emission monitoring technologies for carbon capture processes.

## 2. Amine Emissions and Monitoring in Carbon Capture Processes

### 2.1. Emission Characteristics of Amines

Currently, the exhaust gas pollutants from large-scale amine-based carbon capture systems are mainly composed of organic amine absorbents and volatile degradation products such as ammonia [27,28]. Organic amines in the exhaust gas are the primary emitted pollutants, while the emission concentrations of other degradation products are at least one order of magnitude lower than those of organic amines. Therefore, organic amine absorbent components are the core targets for emission control. Common organic amines used in carbon capture absorption processes include MEA, MDEA, DEA, PZ, AMP, etc. [29,30,31]. At the exhaust gas outlet, the temperature ranges from 20 to 50 °C, the relative humidity (RH) ranges from 70% to 100%, and the interfering substances include NOx and SOx at concentrations below 20 ppm.

Organic amines are mainly emitted in three forms [32,33]. (1) Physical entrainment: Liquid droplets of absorbents larger than 100 μm are carried out by flue gas during intense local contact and collision between gas and liquid phases, with emission concentrations below 1 ppm. (2) Gaseous emission from absorbent volatilization, with concentrations ranging from 1 to 100 ppm. (3) Aerosol emission: An aerosol is a colloidal dispersion system consisting of particles with sizes between 1 nm and 100 μm suspended in a gaseous medium. This type of emission refers to pollutants carried by liquid or solid particles in the system, with concentrations of 10 to 1000 ppm.

Droplets from physical entrainment have large particle sizes. Conventional wire mesh demisters achieve a removal efficiency of over 99% for particles ≥ 10 μm, and such droplets tend to settle naturally. Hence, this fraction of emissions is generally negligible in engineering practice. Aerosol particles smaller than 3 μm are difficult to eliminate, but they can be monitored and controlled using mature conventional particulate matter detection technologies such as PM2.5 monitoring [34].

Gaseous emissions follow the principle of gas–liquid equilibrium. When flue gas comes into direct contact with liquid absorbents inside the absorber, part of the absorbent evaporates into the gas phase, is carried away by the flue gas, and eventually forms gaseous organic amine emissions. The concentration of gaseous organic amines at the absorber outlet is mainly governed by three factors: flue gas temperature, absorbent composition, and the activity coefficient of the absorbent. A higher flue gas temperature leads to a greater partial pressure of the absorbent in the gas phase and a corresponding increase in gaseous emissions. The volatility of typical amine absorbents in ascending order is: DEA < MDEA < PZ < MEA < AMP [35].

The absorbent is an electrolyte system and cannot be regarded as an ideal solution. The absorption reaction between the absorbent and flue gas inside the absorber is exothermic, resulting in a sharp temperature gradient along the tower height and a notable temperature difference between the gas and liquid phases. For multicomponent absorbents, individual organic amine components exhibit distinct differences in volatility. In addition, accurate solution replenishment is difficult to achieve at industrial sites, causing substantial fluctuations in absorbent concentration. These non-ideal characteristics of actual operating conditions make simplified thermodynamic models based on activity coefficients incapable of accurately describing the gas–liquid equilibrium behavior of the system.

### 2.2. Detection Technologies for Amine Emissions

Offline analytical techniques dominate the detection of organic amine emissions during carbon capture processes, with gas chromatography-mass spectrometry (GC-MS) as the representative method [36,37]. This technique enables accurate separation, qualitative and quantitative analysis of volatile by-products. However, it involves complicated sample pretreatment procedures, including derivatization and concentration, leading to a long analysis cycle. Conventional offline methods fail to meet the requirements for real-time monitoring. They cannot capture dynamic variations in emissions or fully characterize the components and flux of organic amine emissions. Accordingly, the development of high-precision real-time online monitoring technologies for organic amines is of great engineering significance and application value.

In contrast, chemical gas sensors, known as “electronic noses” that outperform the limits of human olfaction, can accurately identify trace chemical molecules at ppb and even ppt levels [38,39,40]. As a fingerprint recognition technology for gas molecules, they play a vital role in numerous fields, including environmental remediation, food safety, industrial production, and medical diagnosis. Gas sensors for organic amines have emerged as a key technology for industrial safety production and carbon emission monitoring, and are expected to address the current challenges of inaccurate detection and incomplete characterization of organic amines in industrial scenarios.

## 3. MOS Gas Sensors for MEA Detection

MEA is the most widely used organic amine in the field of carbon capture. Its gaseous leakage poses prominent toxic effects on the human respiratory system and skin. However, research on MEA gas sensors is still relatively scarce. A variety of conductive semiconductor materials have been developed and applied in chemical gas sensors, such as metal oxides [41], organic semiconductors [42], conductive polymers [43], and ion-conducting materials [44]. Among them, metal oxide semiconductor (MOS) gas sensors have become the mainstream technical route for MEA detection due to their low cost, fast response and easy integration. However, there are still some issues to be addressed in MEA detection materials, such as low response and poor selectivity [45]. Common strategies to enhance performance include heterojunction formation [46,47,48,49], elemental doping [50,51,52], composites with carbon materials [53,54], and morphology and surface engineering [55,56,57,58].

Common MOS materials mainly include AOx-based materials, which are the most widely adopted n-type semiconductor substrates for MEA gas sensors. The gas sensing mechanism of AOx relies on oxygen adsorption–desorption processes on the semiconductor surface. Oxygen molecules adsorb onto the material surface and capture conduction band electrons, which thickens the surface electron depletion layer and increases the sensor resistance. When MEA molecules undergo redox reactions with surface-active oxygen, electrons are released back into the conduction band, thinning the electron depletion layer and lowering the sensor resistance. Real-time monitoring of resistance variations enables accurate identification of MEA molecules and their concentration changes, laying a technical foundation for quantitative detection of MEA. In addition, ABO_3_-based semiconductor sensors have also been reported for MEA detection. Although they exhibit fast response, their poor stability in high-humidity environments makes them difficult to apply in carbon capture industrial installations.

In recent years, constructing micro-nano structures such as porous architectures, rough surfaces, and heterojunctions has become an effective sensitization strategy to further enhance the rapid adsorption and recognition of MEA molecules by sensing materials. On the one hand, porous channels create a large specific surface area and abundant porosity, and construct a tailored chemical microenvironment on pore walls. This facilitates the rapid adsorption, mass transfer, and diffusion of MEA molecules, thereby improving gas sensing response and selectivity. On the other hand, the introduction of active components, including noble metal nanoparticles and oxygen-containing groups, provides abundant active sites. Noble metal nanoparticles can exert typical electronic sensitization and chemical sensitization effects, as well as the hydrogen spillover effect, which markedly boosts the reaction activity of MEA molecules and optimizes gas sensing performance. This research direction is expected to deliver new technologies for the precise identification of MEA and in situ real-time monitoring of MEA concentration in industrial reactors.

### 3.1. AOx-Based Materials for MEA Detection

Common AOx materials include ZnO, SnO_2_, In_2_O_3_, and WO_3_, etc. As substrates for gas sensors, their intrinsic electronic structure, surface chemistry, and ability to adsorb/desorb gas molecules directly determine sensor performance. This study systematically evaluates the sensing performance of various pure MOS substrate materials toward MEA based on the existing literature, aiming to clarify the inherent advantages and limitations of each material without complex modifications, thereby providing theoretical guidance for future material selection and design of MEA sensors.

ZnO has attracted attention due to its low cost and ease of preparation. The MEA sensing performance of ZnO materials has been widely studied [59,60,61,62,63]. By using various synthesis strategies such as the hydrothermal method and solvothermal method, pure ZnO materials with different morphologies (such as nanorods, nanoflowers, hollow microspheres, etc.) and microstructures can be prepared. The diversity of these morphologies and structures provides a broad space for optimizing sensing performance. However, high operating temperatures, limited selectivity, variations in response values, and potential humidity interference are the main challenges currently faced by pure ZnO sensors.

SnO_2_ holds a significant position in the field of gas sensing due to its excellent physical and chemical properties as well as electrical performance [64,65,66,67]. The SnO_2_ sensor demonstrates significant advantages in the field of MEA detection, mainly manifested in its high sensitivity (reaching ppb-level detection limits through doping and composite materials), excellent selectivity (having specific recognition ability for MEA molecules), rapid response/recovery time (achievable within seconds after optimization), and good stability and repeatability. However, SnO_2_ sensors also have some inherent drawbacks, including a higher optimal working temperature (usually above room temperature, increasing energy consumption), susceptibility to environmental humidity (humidity increase leads to a decrease in response value), relatively low response values of pure materials, and the tendency for nanoparticles to aggregate. These drawbacks can be effectively overcome through strategies such as composites with other materials (such as Ag, Pd, CsPbBr_3_, g-C_3_N, Ti_3_C_2_Tx), doping with precious metals, or optimizing the morphological structure, thereby further enhancing the comprehensive performance and practical application potential of SnO_2_-based sensors in the MEA detection field.

The MoO_3_-based sensors exhibit remarkable advantages such as high sensitivity, low detection limit, rapid response, excellent selectivity, good repeatability, and long-term stability in MEA detection, and particularly have great potential in room-temperature operation [68,69]. This is attributed to the strong acid-base attraction between MoO_3_ and MEA molecules, as well as the easily breakable C-N bonds and abundant hydrogen bonds of the MEA molecules. However, the performance limitations of pure MoO_3_ and the influence of humidity on the sensor performance are aspects that need further improvement.

In_2_O_3_ and WO_3_ have also been reported. According to the report in Sui’s work [70], the response value of the In_2_O_3_ sensor to 100 ppm MEA at an operating temperature of 220 °C is approximately 25. The research points out that In_2_O_3_ sensors usually have disadvantages such as high operating temperature, insufficient selectivity, and slow response/recovery kinetics, which are mainly attributed to their limited surface-active sites and suboptimal electronic properties. Cao et al. evaluated the MEA sensing performance of WO_3_ material [70]. The study found that the WO_3_ sensor had a response value of only 2.86 to 100 ppm MEA at an operating temperature of 210 °C. The response time was 60 s and the recovery time was 10 s. Compared with SnO_2_ and In_2_O_3_, pure WO_3_ has lower sensitivity to MEA and a slower response speed, but a faster recovery time.

Compared with AOx materials, relatively few studies have reported ABO_3_ perovskite-based semiconductor sensors for MEA detection. Although such perovskite sensors respond faster than their AOx-based counterparts, they suffer from obvious response attenuation during long-term testing, a short service life, and poor adaptability to high-humidity environments, which severely limits their practical deployment. For example, Lin et al. [71] fabricated porous LaFeO_3_ nanofibers with different Ni doping ratios via electrospinning. The sensor exhibited an optimal operating temperature of 260 °C, with a response value of 8.3 toward 100 ppm MEA. Its response time was merely 3 s, and its recovery time was 13 s. In addition, Li et al. [59] successfully prepared gas sensors based on ZnO/NdFeO3 n-p heterojunction composites through a two-step hydrothermal method. The optimal operating temperature of this sensor decreased to 240 °C, and its response value to 100 ppm MEA increased to 25, which was 1.32 times that of the pure ZnO sensor and 1.6 times that of the pure NdFeO_3_ sensor. The response time and recovery time were 19 s and 49 s, respectively, enabling rapid response to variations in MEA concentration. After 21 days of continuous stability testing, the fluctuation of sensor response was less than 10%; the response value remained basically stable at an RH of 5–15%, while it decreased gradually when the RH exceeded 20%. Perovskite materials have a relatively low matching degree with carbon capture application scenarios, making it difficult to promote them in industrial applications.

### 3.2. Morphology Engineering of Sensing Materials

The gas sensing performance is closely related to the nanostructure of the material. By manipulating the morphology, size and microstructure of the material, the specific surface area, the number of active sites, the carrier transport efficiency, as well as the gas adsorption/desorption kinetics can be significantly influenced, thereby optimizing the response, selectivity, operating temperature, and stability of the sensor.

Chao et al. synthesized ZnO hollow microspheres with a particle size of 1.5–2 μm via a carbon microsphere-assisted impregnation and annealing method [62]. The shell of the microspheres was self-assembled from nanoparticles, and Au nanoparticles were uniformly loaded on the surface of ZnO hollow microspheres. Chemical gas sensors were fabricated using the as-prepared hollow microspheres as sensing materials. The 2% Au/ZnO sensor exhibited a response value of 68 toward 100 ppm MEA, which was 4.7 times that of the pure ZnO sensor. It delivered excellent sensing performance for MEA, with a limit of detection as low as 200 ppb. The response time and recovery time for 500 ppb MEA were 13 s and 24 s, respectively. No obvious decay in response was observed after a 100-day continuous stability test. Within the RH range of 10% to 90%, the sensor response slightly decreased with rising humidity but remained at a relatively high level. Such a sensitization effect is primarily attributed to the electronic and chemical sensitization of Au nanoparticles. The exposed active sites can reduce the reaction energy barrier of MEA, and thus enhance the response speed and sensitivity of the sensor.

**Figure 2:**
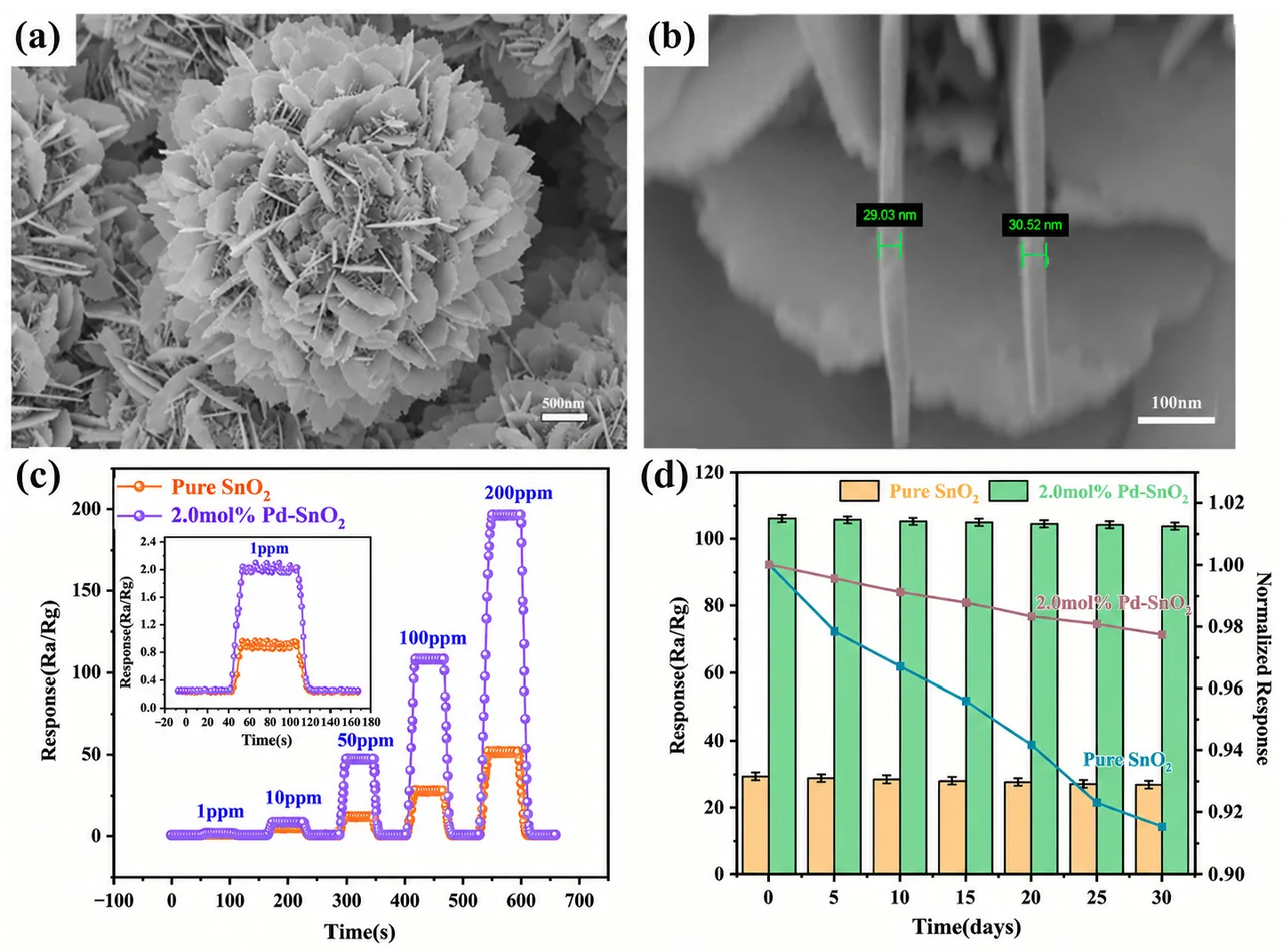
(**a**,**b**) FESEM images of 2.0 mol% Pd-SnO_2_. (**c**) Response/recovery curves of pure SnO2 and 2 mol% Pd-SnO_2_ gas sensors to 1–200 ppm MEA. (**d**) Long-term stability of pure SnO_2_ and 2 mol% Pd-SnO_2_ gas sensors to 100 ppm MEA at 150 °C [67].

Similarly, Dai et al. [60] prepared gas sensors based on Au-modified ZnO rod-like nanoflower composites by combining the hydrothermal method with ion sputtering. The sensor achieved a high response value of 138 to 100 ppm MEA at an optimal operating temperature of 210 °C, with a response time of 15 s and a recovery time of 34 s. It featured fast response, favorable linear correlation, outstanding selectivity, and high stability. Apart from active Au nanoparticles that generate plasmonic effects, other noble metal particles with high reactivity, including Pd, Pt, Ag, and Rh, also exert remarkable sensitization effects. For instance, Bi et al. [67] synthesized Pd-doped SnO_2_ flower-like hierarchical structures via the solvothermal method (Figure 2). The three-dimensional flower-like architecture was self-assembled from two-dimensional nanosheets, possessing a large specific surface area, abundant channels and high structural stability, which greatly improved the adsorption and diffusion efficiency of gas molecules. The sensor worked optimally at 150 °C, showing a sensitivity of 106 toward 100 ppm MEA, a response time of 10 s and a recovery time of 12 s, with a limit of detection of 1 ppm. Approximately 8% attenuation in response was recorded after a 30-day continuous stability test.

### 3.3. Modification Strategies for Sensing Materials

Furthermore, multicomponent metal oxide materials can largely compensate for the deficiencies of single-component materials in terms of active site types and reaction interfaces, and realize synergistic sensitization, thereby achieving superior sensing performance. As a typical case, Ding et al. [72] synthesized ZnO/In_2_O_3_ composite gas sensing materials using bimetallic metal-organic frameworks (MOFs) as precursors through the hydrothermal method. At the operating temperature of 240 °C, the sensor presented an ultra-high response value of 237 to 50 ppm MEA. A desirable linear correlation between sensor response and MEA concentration was obtained in the range of 1–50 ppm. The response time was 31 s and the recovery time was 21 s. After a 30-day long-term stability test, the response attenuation was less than 5%, and no significant drift of baseline resistance was observed.

Besides noble metal doping and multicomponent hybridization strategies, constructing heterojunction interfaces is a preferred technique to boost performance in sensing and catalysis. Integrating materials with different properties or functions to form heterogeneous interfaces can generate abundant active sites and enhance adsorption capacity at the interfaces.

**Figure 3:**
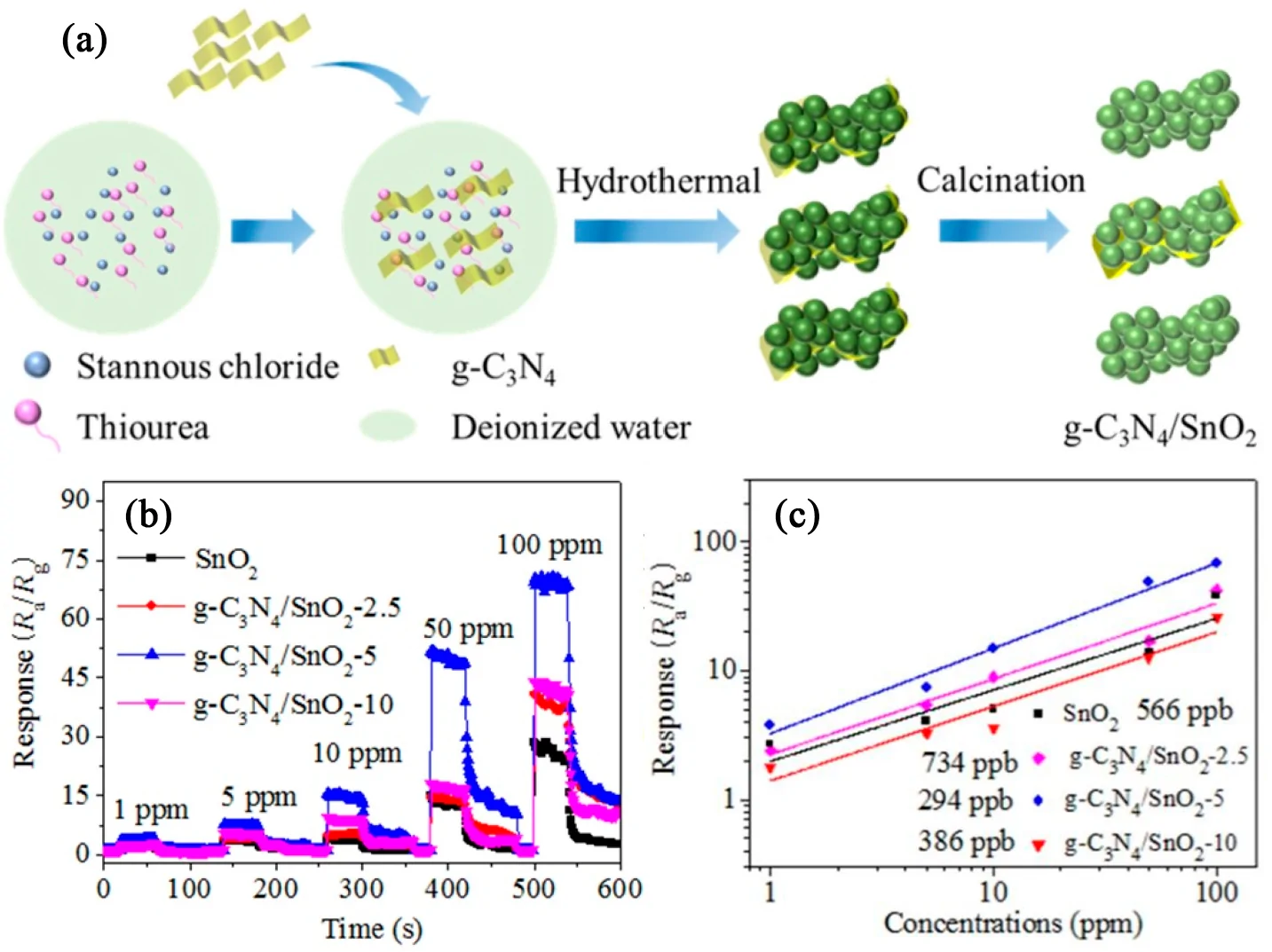
(**a**) Schematic diagram of the formation process. (**b**) Transient response–recovery profiles of composite material at 240 °C after exposure to different concentrations of MEA. (**c**) Linear relationships between response and concentration of MEA [66].

For example, Xu et al. [65] innovatively combined two-dimensional transition metal carbides (MXene) Ti_3_C_2_Tx with tin dioxide (SnO_2_) to fabricate an n-n type Ti_3_C_2_Tx/SnO_2_ heterojunction gas sensor. This sensor exhibits high response, low limit of detection, excellent selectivity toward hydrogen-bonded gases, and long-term stability for MEA. Its optimal operating temperature is 160 °C. A favorable linear correlation exists between the response value and MEA concentration within the low-concentration range of 1–15 ppm. The response time is 51 s, and the recovery time is 167 s. After 30 days of continuous testing, the fluctuation of the sensor response is less than 2%, with no obvious drift in baseline resistance. The sensor delivers the best performance at 38% RH. When RH rises from 38% to 68%, the response value drops from 20 to 4.5. Rich -OH and -O functional groups on the surface of Ti_3_C_2_Tx can form strong hydrogen bonds with MEA molecules, enabling specific recognition of hydrogen-bonded gases. Similarly, Li et al. [66] synthesized n-n type g-C_3_N_4_/SnO_2_ heterojunction gas sensing materials via a two-step method combining urea pyrolysis and hydrothermal treatment, achieving uniform dispersion of SnO_2_ nanoparticles on g-C_3_N_4_ nanosheets (Figure 3). Benefiting from the Type II band alignment structure, the material facilitates interfacial electron transfer and oxygen adsorption, and produces more reactive oxygen species to intensify the redox reaction with MEA molecules, which greatly improves the response speed and sensitivity of the sensor. The optimal operating temperature is 240 °C. The response value shows a good linear relationship with MEA concentration in the range of 1–100 ppm. For 100 ppm MEA, the response time is only 1 s and the recovery time is 20 s, making it one of the fastest-response MEA gas sensors reported so far.

**Figure 4:**
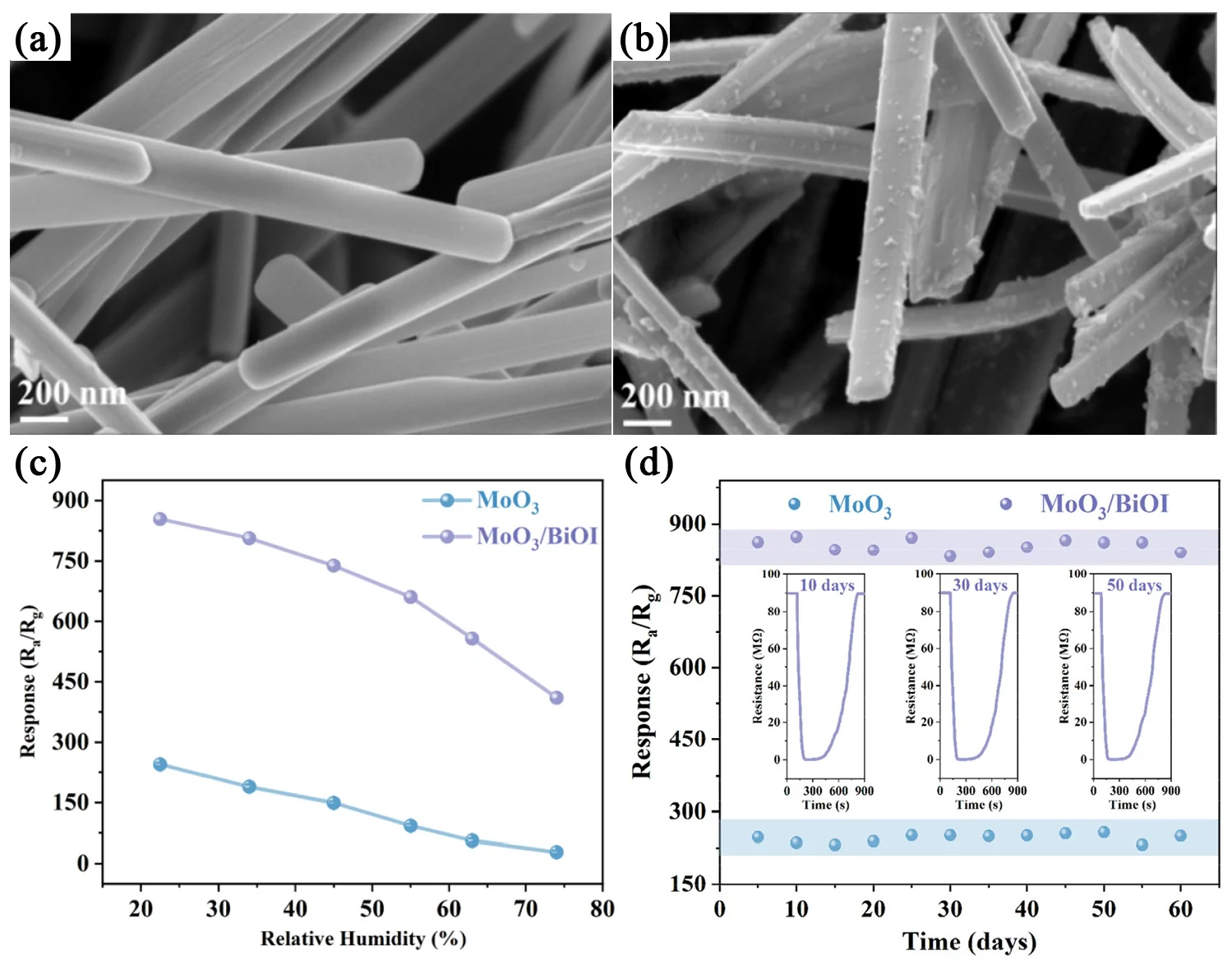
(**a**) SEM image of MoO_3_. (**b**) SEM image of MoO_3_/BiOI. (**c**) Response values of MoO_3_ and MoO_3_/BiOI to MEA under different RH. (**d**) Long-term stability of MoO_3_ and MoO_3_/BiOI [68].

In addition to n-n heterojunctions, other common heterojunction structures such as p-n, p-p, and p-n-p heterojunctions can also deliver prominent sensing activity. Recently, Zheng’s group [68] constructed n-type MoO_3_ nanobelt/p-type BiOI nanoparticle heterojunctions by combining the hydrothermal method with cyclic impregnation (Figure 4). For the first time, ultra-sensitive detection of MEA was realized at room temperature, and the sensor achieved an extremely high response value of 861.3 toward 100 ppm MEA. In the concentration range of 0.5–100 ppm, the response value increases remarkably with the rise of MEA concentration and complies well with a quadratic function. A good linear correlation is observed in the low-concentration range of 0.5–7 ppm. For 100 ppm MEA, the response time is 56 s and the recovery time is 349 s. The response value is 861 at 25% RH and remains as high as 396 when RH increases to 75%, demonstrating satisfactory humidity resistance. After 60 days of continuous stability testing, the response fluctuation is less than 5%, which proves its outstanding long-term stability and repeatability.

Considering that both the sensitization effect of noble metals and heterojunction interfaces can significantly enhance the reactivity of sensing materials toward MEA, researchers have combined these two sensitization strategies. The synergistic effect of noble metals and heterojunction structures further improves the sensing activity of substrate materials and achieves a more prominent sensitization performance. For instance, Xu et al. [64] fabricated ternary nano-heterojunction gas sensing materials consisting of Ag nanoparticle-modified SnO_2_ and 3-mercaptopropionic acid (MPA)-stabilized CsPbBr_3_. This work realized efficient room-temperature detection of MEA using SnO_2_-based sensors for the first time. At room temperature, the maximum response value of 2Ag@SnO_2_/CsPbBr_3_ to 100 ppm MEA reached 43, which was seven times that of the pure CsPbBr_3_ sensor and 2.3 times that of the SnO_2_/CsPbBr_3_ heterojunction sensor. When the operating temperature increased to 50 °C, the response value decreased to 8.65. A favorable linear correlation was observed within the low-concentration range of 5–15 ppm. The response time was 45 s, and the recovery time was 684 s. The sensor showed a response value of 40 at 18% RH, which dropped to 15 when RH rose to 60%, indicating that the sensor is more suitable for low-humidity environments. After a 30-day continuous stability test, the fluctuation of the sensor response was less than 8%, demonstrating excellent long-term stability.

### 3.4. Current Problems and Expected Solutions

As summarized in Table 1, AOx-based semiconductor sensors generally show satisfactory sensitivity and response time for MEA detection under laboratory conditions, which is close to the requirements of current carbon capture technologies. The main obstacle to industrial implementation is therefore not the response magnitude itself, but the mismatch between laboratory evaluation and real flue gas conditions. Most studies rarely test sensors at RH above 80%, so the effect of abundant water vapor in industrial flue gas remains poorly understood. The interfering species considered are usually limited to organic compounds such as ethanol, acetone, toluene, formaldehyde, methanol, and dichloromethane, while SOx, NOx, and dust, which are common in actual operation and can corrode gas lines, react with amines, and deposit on sensing surfaces, are seldom examined. In addition, many sensors require high operating temperatures, and high-temperature sampling may cause volatilization, oxidation, or decomposition of MEA, especially in the presence of oxygen and acidic gases, leading to systematic measurement deviations. Finally, the MEA concentration in exhaust gas is usually relatively stable, and the ultra-low VOC emission limit is around 20 ppm, whereas most studies evaluate response using a 100 ppm step gas, which cannot reflect performance under steady low-concentration flow.

Judged by these criteria, ZnO-based sensors are difficult to apply directly, because their operating temperatures mostly fall between 210 and 300 °C and their humidity data rarely cover the wet flue gas region. MoO_3_, In_2_O_3_, and WO_3_ are limited by slow recovery, relatively high detection limits, or insufficient evidence of humid stability, and none of them has been systematically validated against SOx, NOx, and dust. SnO_2_-based composites are more promising, since noble metal sensitization, coupling with carbonaceous materials or graphitic carbon nitride, heterojunction construction, and oxygen vacancy engineering can reduce the activation energy of MEA adsorption and catalytic oxidation, allowing operation around 150 °C or even at room temperature, which limits thermally induced MEA loss and suits low-power devices. For humidity, hydrophobic breathable coatings and molecular sieve encapsulation are more reasonable solutions than simple composition tuning, because they can hinder water molecules while leaving diffusion channels for target gases. A practical configuration for industrial CCUS should therefore combine a low-temperature SnO_2_ composite with a hydrophobic membrane or a selective filtration and pretreatment unit that removes water vapor, SOx, NOx, and dust, together with temperature and humidity compensation and pattern recognition to correct residual cross-interference.

Future work should move from single-material optimization to the integration of materials, devices and algorithms. On the material side, single-atom catalysis combined with heterojunction and defect engineering can improve the utilization of precious metals and shorten the slow desorption and long recovery that commonly occur at room temperature. Controlling exposed crystal planes, pore size distribution and shell thickness allows multidimensional structures such as 0D quantum dots, 1D nanowires and 2D nanosheets to be assembled, optimizing adsorption sites, gas diffusion, and electron transport while improving long-term structural stability. On the device side, heat tracing, dehumidification, filtration, and selective permeation membranes should not only be evaluated for interference removal, but also for their effects on MEA recovery and response delay. On the algorithm side, sensor arrays combined with pattern recognition can compensate for drift and cross-sensitivity. The evaluation protocol should also shift from 100 ppm step gas to stable low-concentration MEA flow containing realistic interferents, and the required detection limit and accuracy should be defined by the end use of CO_2_, since food-grade, medical-grade, and synthetic fuel pathways are much more sensitive to impurities than enhanced oil recovery or mineralization.

## 4. Conclusions

Carbon capture technology based on chemical absorption with organic amines is the most technically mature and widely applied core route in the current CCUS system. During large-scale industrial operation, the combined gaseous volatilization and emission of organic amine absorbents not only leads to absorbent loss and reduced capture efficiency, but also causes secondary environmental pollution and public health risks. The required CO_2_ purity and monitoring stringency also depend on the utilization pathway, since trace amines are intolerable in agricultural, food, and chemical synthesis applications. Accordingly, developing high-precision real-time online monitoring technologies for organic amine emissions is essential to ensure the safe and stable operation of CCUS systems, control secondary pollution risks, and advance the green and low-carbon development of the industry.

MOS gas sensors have become the mainstream technical route for MEA detection due to their distinct advantages, including low cost, fast response, and easy miniaturization and integration. Relevant studies mainly focus on AOx-based materials, among which ZnO and SnO_2_ are typical semiconductor representatives. Strategies such as precise morphology regulation, noble metal catalytic modification, and heterojunction interface engineering can effectively tailor their surface oxygen vacancy concentration and electron transport properties, thereby greatly improving the gas sensing sensitivity, selectivity, and long-term stability toward MEA. Among them, SnO_2_-based multicomponent composites enable efficient detection at low temperatures and even room temperature, which meets the monitoring requirements for low-concentration emissions in industrial scenarios. ZnO-based materials also deliver excellent gas sensing performance via the design of hierarchical structures such as hollow microspheres and nanoflowers. By contrast, ABO_3_-type perovskite materials exhibit high sensitivity but suffer from poor long-term operational stability and severe performance degradation under high-humidity conditions, posing challenges to their practical engineering application.

Although existing MOS sensors can achieve efficient detection of MEA under laboratory conditions, there is a notable disconnect between current research findings and the practical monitoring requirements of amine-based carbon capture facilities. Most studies are conducted under ideal laboratory environments with low humidity and single interfering components, lacking systematic performance verification under complex industrial flue gas conditions, such as high relative humidity (>80% RH) and the presence of SOx, NOx, and dust. The optimal operating temperature of most sensors exceeds the thermal degradation and oxidation temperature of organic amines, including MEA (140 °C), which tends to decompose the target gas and cause systematic measurement errors. In addition, performance evaluation is mostly performed using a high-concentration MEA of 100 ppm as the benchmark. Few investigations focus on the response characteristics and long-term reliability for actual stable low-concentration emissions (≤20 ppm) in industrial settings, which greatly hinders the progress of engineering application.

The research and development of MOS gas sensors should focus on SnO_2_-based multicomponent composites and four core objectives—low-temperature detection, high-humidity resistance, strong anti-interference capability, and long-term stability—so as to promote the transformation of relevant technologies from laboratory research to industrial application. Future research shall focus on the following aspects:

(1) Further explore heterojunction band engineering and surface defect engineering to precisely regulate the band structure and distribution of surface-active sites of composite functional materials, and break through the performance bottlenecks under low-temperature (≤140 °C) and high-humidity conditions.
(2) Establish standardized test platforms that closely simulate actual industrial scenarios, systematically clarify the influencing mechanisms of water vapor, acid gases, and dust on sensor responses, and formulate evaluation methods for long-term reliability.
(3) Make breakthroughs in key technologies for sensor arraying, miniaturization, and low-power integration, develop online monitoring terminals for organic amine emissions, and build an intelligent management platform integrating monitoring, early warning, and source tracing. Pilot tests and industrial demonstration applications should also be carried out.

Through multi-technology collaboration and system integration, the emission of organic amines can be minimized while guaranteeing CO_2_ capture efficiency. This will drive the advancement of amine-based carbon capture technology toward environmental friendliness, safety and sustainability, and provide core support for the large-scale and green development of CCUS technologies.

## Figures and Tables

**Table 1:** Quantitative comparison table of MEA detection.

Materials	Morphology	Modification	Response Ratio (Ra/Rg or Rg/Ra)	LOD	Response/ Recovery Time, s	RH Range	Operating Temperature
ZnO [62]	hollow spheres	Au	4.7	200 ppb	13/24	10–90%	240 °C
ZnO [60]	rod-like	Au	1.7	1000 ppb	15/34	Not reported	210 °C
ZnO [56]	Not reported	In_2_O_3_	1.4	1000 ppb	31/21	<65%	240 °C
ZnO [59]	Not reported	NdFeO_3_	1.6	Not reported	19/49	<30%	240 °C
ZnO [50]	Not reported	S	4	89 ppb	1/40	Not reported	240 °C
ZnO [61]	flower-like	pH value	4~20	140 ppb	1/195	<60%	220 °C
ZnO [63]	porous	biomass	2.1	Not reported	26/2	Not reported	300 °C
SnO_2_ [67]	flower-like	Pd	3.6	1000 ppb	10/12	Not reported	150 °C
SnO_2_ [65]	2D	Ti_3_C_2_Tx	1.9	76 ppb	51/167	<68%	160 °C
SnO_2_ [66]	2D	g-C_3_N_4_	2.6	294 ppb	1/20	Not reported	240 °C
SnO_2_ [64]	Not reported	Ag, CsPbBr_3_	7	44 ppb	45/684	<60%	RT
MoO_3_ [68]	Not reported	BiOI	3.5	130 ppb	56/349	<75%	RT
MoO_3_ [69]	2D	Ti_3_C_2_Tx	1.6	Not reported	5/9	<80%	180 °C
In_2_O_3_ [70]	nanofibers	Zn	3.7	647 ppb	7/10	Not reported	200 °C
WO_3_ [56]	popcorn-shaped	yeast	2.4	5000 ppb	30/6	Not reported	180 °C

## Data Availability

No new data were created or analyzed in this study. Data sharing is not applicable to this article.

## Abbreviations

The following abbreviations are used in this manuscript:

MDEA	N-MethyldiMEA
DEA	DiMEA
PZ	Piperazine
AMP	2-Amino-2-methyl-1-propanol
